# Predictors of mental health symptomatology among Kurdish patients who recovered from COVID-19 in Iraq

**DOI:** 10.1007/s44202-022-00043-5

**Published:** 2022-06-27

**Authors:** Hawkar Ibrahim, Katharina Goessmann, Araz Ramazan Ahmad, Ayoub Kareem Saeed, Frank Neuner

**Affiliations:** 1 Department of Psychology, Clinical Psychology and Psychotherapy, Bielefeld University, P.O. Box 10 01 31, 33501 Bielefeld, Germany; 2 vivo International, Konstanz, Germany; 3 College of Humanities, University of Raparin, Ranya, Iraq; 4 Sulaimani Polytechnic University, Sulaymaniyah, Iraq

**Keywords:** COVID-19, PTSD, Depression, Mental health, Hospitalization

## Abstract

While a growing body of research has documented severe psychosocial consequences of the new Coronavirus disease (COVID-19) for the affected people, research mainly focused either on health care workers or the general population. There is a dearth of scientific research on the mental health status of recovered patients, especially in low- and middle-income countries. The purpose of the current study was to determine the mental health symptomatology and its associated factors among Iraqis who recovered from COVID-19. Participants were Iraqi Kurdish individuals who had previously been diagnosed with COVID-19 during the first wave of COVID-19, and they were recruited based on lists of recovered patients provided by from public health institutions. Using standardized demographic and mental health questionnaires, structured telephone interviews with 57 recovered patients were contacted. It was found that 31.6% of the participating recovered patients with COVID-19 met the diagnostic criteria for post-traumatic stress disorder (PTSD), and 21.05% were classified with probable depression. Hospitalized survivors had higher PTSD and depression symptoms than those not hospitalized. We also found that higher levels of PTSD and depression symptoms were predicted by younger age, hospitalization due to COVID-19, and having a family member who died from COVID-19. In the context of the global COVID-19 pandemic, psychological and psychiatric treatment may be particularly relevant for younger adult patients and those with more severe COVID-19 symptoms who experienced hospitalized care.

## Background

A new coronavirus disease (COVID-19) in 2019 resulted in one of the largest-scale worldwide outbreaks of infections in modern history. Evidence from previous health pandemics and epidemics, such as Severe Acute Respiratory Syndrome (SARS), Middle East Respiratory Syndrome (MERS), and Ebola virus disease, has suggested that the outbreak’s negative impacts are not limited to affecting one’s physical health or economic, social, and political disruption, but there has also been a mental health toll, as well [1–4].

Consistent with findings from previous epidemics, several studies have investigated the potential mental health impacts of COVID-19, reporting various mental health issues including mood, anxiety, insomnia, and post-traumatic stress disorder (PTSD), and they identified various risk factors for negative mental health outcomes in affected populations [5–11].

While these studies provide significant pathways in understanding and treating mental health conditions among affected people, considerable limitations exist in researching health sequelae of COVID-19. First, the research in this area focuses mainly either on health care workers or the general population [12–15]. Little attention has been paid to potential mental health impacts among people who recovered from COVID-19. Moreover, almost all the mental health studies that recruited patients with COVID-19 were either conducted during their period of hospitalization or in the post-vaccination era; knowledge about the mental health status of recovered patients in the early stages of the COVID-19 outbreak and prior to vaccination has been scarce [16]. Second, the vast majority of these studies have been conducted in high-income Western countries and China; data documenting the mental health consequences of COVID-19 in low- and middle-income countries (LMICs) are lacking. Due to socioeconomic inequities, war and political conflict, and poor-quality health care in many LMICs, it's unsuitable to transfer and generalize mental health findings from high-income and Western countries to the LMICs.

It's well known that social factors on both individual (i.e., age, gender, race, ethnicity, marital status) and societal levels (i.e., kinship, familial and social relationships) greatly influence individuals' mental health [17–19]. Such influences may be higher in collective societies such as Iraq where family and social relationships significantly shape individuals' well-being [20]. Therefore, it is plausible to expect tremendous suffering in terms of mental health symptomology among individuals whose family members were also affected or died unexpectedly due to COVID-19 infections. However, this notion remains understudied, and scientific evidence of the role of potentially traumatic quality of the consequences of COVID-19 diseases, such as hospitalization or familial impacts, is limited [16, 21]. These understudied factors are crucial elements in increasing cross-cultural knowledge of the psychological effects of COVID-19, especially in collective and LMICs.

The present study attempts to fill the above-mentioned gaps, and it’s aimed to understand the mental health symptomatology and its associated factors of Iraqis who survived COVID-19 infection in the early phase of the pandemic. In particular, we aimed to estimate prevalence rates of PTSD and depressive disorders using valid and reliable screening tools among Iraqi patients who had recovered from COVID-19. For this purpose, we drew a small sample based on recovered patients lists from public health institutions who are managing COVID-19 patients in northern Iraq.

## Methods

### Participants and procedures

The participants in this study were Iraqi Kurdish individuals who had previously been diagnosed with COVID-19 during the first wave of COVID-19. They were identified based on data (list of COVID-19 recovered patients between August and October 2020) available through the General Health Directorate of Raparin in the northern Iraq.

Inclusion criteria were as follows; (1) age 18 or older; (2) previously diagnosed with COVID-19 based on a polymerase chain reaction (PCR) test; (3) with no reported and registered psychiatric history; (4) fully recovered from COVID-19 for at least one month and confirmed by a negative lab-based PCR test. In total, sixty-two individuals meet the above criteria. Five of the 62 persons contacted declined participation, so the response rate was 91.94%. Due to COVID-19 movement restrictions and for safety protection of participants and researchers, telephone semi-structured rather than face to face interviews were conducted in the Kurdish-Sorani language.

During the interview, participants were asked about their socio-demographic information (e.g., gender, age, marital status, education), health issues (i.e., having chronic physical and psychiatric diseases, hereditary conditions/diseases) and experiences regarding COVID-19 (such as hospitalization due to COVID-19, length of stay in hospital, and number of family members affected by COVID-19) as well as depression and PTSD symptoms, which were assessed using valid and reliable Kurdish Sorani versions of the Depression Scale of the Hopkins Symptom Checklist-25 (DHSCL-25) and the PTSD Checklist for DSM-5 (PCL-5) [22, 23]. PCL and DHSCL are the most widely applied measures for assessing PTSD and depression in cross-cultural research [24, 25]. The PCL-5 contains twenty items rated on a five-point Likert-type scale, with scores ranging from “Not at all” (0) to “Extremely” (4), resulting in a symptom severity score between 0 and 80. The DHSCL-25 is designed to assess the presence and severity of depressive symptoms on 15 items scored from 1 (not at all) to 4 (extremely). In the current study, both PCL-5 and DHSCL-25 demonstrated excellent internal consistency (Cronbach's αs = 0.80). Having been diagnosed with COVID-19 was defined as a stressor criterion for PTSD.

All 57 study participants were Iraqi Kurdish patients who had recovered from COVID-19. Nearly three quarters of participants were male (73.7%). The mean age was 38.39 years (*SD* = 11.55; range 18–80). The vast majority of participants (84.2%) were married, and they had completed between 0 and 19 years of formal education (*M* = 11.21, *SD* = 5.33). At the time of interview, nine of the 57 participants (15.8%) reported experiencing chronic diseases.

Almost two-thirds of participants (63.2%) had at least one family member who was also affected by COVID-19 (*M* = 3.72, *SD* = 5.65; range 1–30), and 17.6% of the total participants had at least one close family member who died as a result of COVID-19 (*M* = 0.19, *SD* = 0.44; range 0—2). One fifth (21.1%) of participants experienced hospitalization due to COVID-19. Hospitalized participants had stayed between 1 to 13 days in hospitals (*M* = 4.66, *SD* = 4.03), and they were aged 18 to 70 years (*M* = 36.58, *SD* = 13.48). While half of the hospitalized participants had been admitted to intensive care units (ICU) for 1 to 4 days (*M* = 2.5, *SD* = 1.64), only 33.33% of them had been on a ventilator.

The study was certified by the Ethics Committee of Koya University in the northern Iraq (Reference number 2020, DPC/02).

### Statistical analysis

Descriptive (means and standard deviations) and frequency statistics were used to describe and summarize the characteristics of participants. The significant difference between demographic groups was calculated using independent samples t-tests. Pearson correlation coefficient was used to estimate the relationship between variables. Multivariate linear regression was utilized to predict potential factors associated with PTSD and depression symptoms. The data were analyzed using IBM SPSS Statistics (Version 28).

## Results

### Levels of mental health symptomatology

The mean sum scores of mental health symptomatology levels were 17.43 (*SD* = 13.96) on the PCL-5 and 21.43 (*SD* = 6.10) on the DHSCL-25. There were no significant gender differences in levels of depression and PTSD symptoms (*P* > 0.05). Using the contextually valid cutoff score of 23 for the PCL-5 and the internationally accepted cutoff score of 1.75 for the DHSCL-25, 31.6% of participants had probable PTSD diagnosis, and 21.1% were classified with probable depression.

### Hospitalization and mental health symptomatology

Participants who had been hospitalized due to a COVID-19 infection reported significantly higher levels of PTSD and depression symptoms compared with those not hospitalized (PTSD: hospitalized; *M* = 28.41, *SD* = 16.10; non-hospitalized; *M* = 14.51, *SD* = 11.90; *p* < 0.01; depression: hospitalized; *M* = 25.91, *SD* = 7.25; non-hospitalized; *M* = 20.24, *SD* = 5.22; *p* < 0.01). Among hospitalized patients, comparisons between ICU and non-ICU patients led to no statistically significant difference in either depression or PTSD symptoms (*p* > 0.05). Also, no associations were found between length of stay in ICU and symptoms of PTSD and depression (*r* = 0.16; *r* = 0.15, *p* > 0.05, respectively).

### Prediction of mental health symptomatology

To examine potential predictors of mental health symptomatology (PTSD and depression symptoms), multivariate linear regression was performed. The sum scores of PTSD and depression were identified as outcome variables, and participants' gender, age, marital status, been hospitalized due to COVID-19, and death of family members due to COVID 19 were introduced as independent variables. As demonstrated in Table 1, results showed that younger age, having a family member who died from COVID-19, and hospitalization due to COVID-19 were significantly associated with higher levels of PTSD and depression symptoms.

**Table 1:** Predictors of mental health symptomatology

Predictors	Depression		PTSD	
	Standardized *ß*-Coefficient	Zero-order correlation	Standardized *ß*-Coefficient	Zero-order correlation
Gender	− 0.007	0.063	0.059	0.105
Age	− 0.285*	− 0.360**	− 0.406**	− 0.401**
Marital Status	− 0.081	− 0.287*	0.052	− 0.219
Hospitalization due to COVID-19	0.263*	0.382**	0.277*	0.410**
Death of family members due to COVID 19	0.294*	0.401**	0.285*	0.395**

Depression = (*F* (5,51) = 5.34, *p* < 0.001), with an *R*^2^ of 0.34. PTSD = (*F* (5,51) = 6.35, *p* < 0.001), with an *R*.^2^ of 0.38

*p < 0.05

**p < 0.01

## Discussion

The present study was designed to determine the mental health symptomatology and associated factors among patients who recovered from COVID-19 in Iraq. For this purpose, a group of Iraqi patients were recruited in this study, and the recruitment was based on recovered lists from public health institutions. The analyses identified high levels of mental ill-health symptomatology with several clinical, social, and demographic predictors. The study thus adds important scientific knowledge on the potential impact of the COVID-19 pandemic in the LMICs.

In line with present systematic reviews and meta-analyses on the psychiatric consequences of coronavirus infections [12, 26], we found high prevalence rates of PTSD and depression among Iraqi patients who recovered from COVID-19. Of the present sample, one third and one fifth presented with symptom levels for probable diagnoses of PTSD and depression, respectively. The rates were comparable with those reported in previous studies [5, 8, 9], and indicated considerable mental strains for the interviewed COVID-19 survivors.

Regarding predictors of mental health symptomatology, consistent with previous findings, we found that hospitalization due to COVID-19 is associated with poor mental health outcomes [27]. Due to having a long history of political violence, economic sanctions, and corruption, Iraqi health sectors have limited capacity to respond to crises such as COVID-19 adequately. Consequently, only high-risk cases were detected and among them only severely affected people were admitted to the hospitals during the first wave of the COVID-19 pandemic [28]. In this context, hospitalization due to COVID-19 may increase the perception about how critical the condition is, which is usually associated with an increased risk of symptoms of mental health disorders [29]. More broadly, hospitalization due to life-threatening conditions is a severe traumatic event and has been strongly linked with mental ill-health symptoms [30].

In addition, we found that the loss of a family member due to COVID-19 increased the risks of developing PTSD and depressive symptoms. Taking the nature of grief in Kurdish culture, which is a family-oriented and collectivist culture, into account, this result is not surprising. Previous research has shown that across all cultural values, the death of a loved one is associated with various mental ill-health symptoms, including but not limited to anxiety, depression, and PTSD [31, 32]. The structure of the Kurdish family is based on tribal traditions [33]. As a result, it has lower relational mobility, meaning that Kurdish family members are emotionally and materially interdependent, and they typically have stable and long-lasting relationships with each other. In this context, losing a family member is highly predicted to bring about intense negative emotional responses such as sadness, sorrow, and other mood symptoms and would thus be associated with high levels of mental health symptoms. Although previous studies have suggested particularly burdened mental health states among COVID-19 patients in intensive care [34–36], ICU admission was not related with higher psychological symptom severity in this sample.

The present results confirmed previous findings and contributed additional evidence suggesting that older adults have higher resilience to the mental health effects of COVID-19 [36–38]. However, given the negative impact of COVID-19-related hospitalization on mental health, a confounding effect of older age with more severe symptom trajectories and more probable hospitalization needs to be taken into consideration regarding older survivors of COVID-19 [16, 39].

Overall, our findings are consistent with previous studies in other populations, which observed considerable rates of mental health symptoms among patients with COVID-19 even after their recovery. Stress-related symptomatology can be an important impairment for general health recovery after illness [35]. In the context of the global COVID-19 pandemic, psychological and psychiatric treatment may be particularly relevant for younger adult patients and those with more severe COVID-19 symptoms who experienced hospitalized care.

The high levels of mental ill-health symptomatology found in this study, shed light on the importance of providing mental health care for Iraqi COVID-19 survivors. Iraqi society has been traumatized by large scale political repression and organized violence, and adequate mental health services in Iraq are lacking [40, 41]. So far, the primary focus of the Iraqi healthcare system is treating and preventing physical rather than mental health issues [42], and trauma-informed care has not been implemented in the healthcare system. The finding of our study calls upon local authorities and international health organizations to scale up the mental health services in Iraq and provide urgent mental health services for survivors of COVID-19.

## Strengths and limitations

The key strength of this study is the sample of full recovery Iraqi COVID-19 patients who had no recorded psychiatric history. Moreover, contextually valid and reliable measurements for assessing mental health symptoms were used and were administered in semi-structured telephone interviews. Nevertheless, caution must be applied with respect to the small sample size, and the findings might not be transferable to other contexts. Moreover, since the prevalence rates of mental health conditions presented in the current study are based on screening tools rather than clinical interviews, the present investigation has not been able to estimate representative prevalence rates of PTSD and depression among COVID-19 patients in Iraq.

While we controlled for the impact of some individual characteristics of the participants, other potential risk factors for mental health problems amidst a pandemic, such as ﻿living situation, financial and job insecurity, social isolation, and lack of support were not assessed [16]. Although 17.6% of the participants in this study had lost at least one close family member, the study did not include information about the role of grief and bereavement on participants’ mental health nor did it control for additional stressors or traumas in the current lives of the interviewees. Likewise, the study also did not assess the severity of COVID-19 symptoms, which may have an impact on prevalence rates of both PTSD and depression [9]. More research with a larger sample including comparisons between infected and non-infected individuals as well as long-term follow-up studies is required to examine the psychosocial consequences of COVID-19 infection in low-resource countries such as Iraq.

## Conclusions

This study examined mental health symptomatology and its associated factors among Iraqi adults who fully recovered from COVID-19. Findings suggest high prevalence rates of COVID-19-related PTSD and depression. Moreover, findings also showed that younger and hospitalized patients, as well as those who lost family members due to COVID-19, are more vulnerable to experiencing more PTSD and depression symptoms.

## Data Availability

Datasets generated from this study will be available from the corresponding author (Hawkar Ibrahim) upon a reasonable request.

## Abbreviations

COVID-19: Coronavirus disease 2019
PTSD: Post-traumatic stress disorder
PCL-5: PTSD checklist for DSM-5
DHSCL-25: Depression Scale of the Hopkins Symptom Checklist-25
